# Quantifying the Role of Lysine in Prion Replication by Nano-LC Mass Spectrometry and Bioassay

**DOI:** 10.3389/fbioe.2020.562953

**Published:** 2020-09-23

**Authors:** Christopher J. Silva, Melissa L. Erickson-Beltran, Irina C. Dynin

**Affiliations:** Western Regional Research Center, United States Department of Agriculture, Agricultural Research Service, Albany, CA, United States

**Keywords:** prion, mass spectrometry, Sc237, lysine, bioassay

## Abstract

Prions propagate by a template driven process, inducing the normal cellular isoform (PrP^C^) to adopt the prion (PrP^Sc^) conformation. In PrP^C^, the positions of lysines are highly conserved and strongly influence prion propagation. In this study, covalent modification was used to quantitate the role of lysines in the PrP^Sc^ template that drives prion replication. The ε-amino group of lysines in the PrP^Sc^ (hamster-adapted scrapie Sc237) template was acetylated by either acetic anhydride (Ac_2_O) or the N-hydroxysuccinimide ester of acetic acid (Ac-NHS). The extent of lysine acetylation in PrP^Sc^ was quantitated by mass spectrometry or Western blot-based analysis. Identical samples were bioassayed to quantitate the loss of infectivity associated with lysine acetylation. The reduction of infectivity at the highest reagent concentration was approximately 90% (∼10-fold). Ten of the eleven prion lysines were acetylated to a greater extent (25−400-fold) than the observed loss of infectivity. Only one lysine, at position 220 (K_220_), had a reactivity that is consistent with the loss of infectivity. Although lysines are highly conserved and play a crucial role in converting PrP^C^ into the PrP^Sc^ conformation, once that conformation is adopted, the lysines present in the PrP^Sc^ template play only a limited role in prion replication. In principle, this approach could be used to clarify the role of other amino acids in the replication of prions and other prion-like protein misfolding diseases.

## Introduction

Prions (PrP^Sc^) are molecular pathogens, which propagate by inducing the normal cellular prion protein (PrP^C^) to adopt the prion’s conformation and, thereby, propagate an infection (Telling et al., 1996; Prusiner, 1998). This template-mediated process is stoichiometric as opposed to catalytic or enzymatically driven. If there is a mismatch between the primary amino acid sequence of the PrP^Sc^ template and the PrP^C^ substrate, then there can be a significant delay in the onset of disease. This delay is traditionally referred to as the species barrier (Pattison, 1965), even though it can occur when both PrP^C^ and PrP^Sc^ are from the same species (Supattapone et al., 1999).

The introduction or deletion of lysines has been shown to significantly affect prion replication in animals. Using transgenic mouse models, researchers showed that deletion of the three N-terminal lysine groups severely impedes prion replication (Turnbaugh et al., 2012). Experimentally infected heterozygous sheep (glutamine/lysine at position 171) succumb to scrapie after a longer incubation period than homozygous (glutamine/glutamine at position 171) sheep (Greenlee et al., 2012). In goats, a lysine at position 222 (K_222_) has also been associated with protection from scrapie infection (Vaccari et al., 2006; Bouzalas et al., 2010). Replacement of a glutamic acid at position 211 with a lysine in bovine PrP^C^ is associated with sporadic disease (Nicholson et al., 2008). These results demonstrate that lysines are crucial for conversion of PrP^C^ to PrP^Sc^.

Analogous results have been observed in the susceptibility of human populations to Creutzfeldt-Jakob disease (CJD). Replacement of glutamic acid at position 196 or 200 with a lysine in PrP^C^ is associated with inherited CJD (Spudich et al., 1995; Peoc’h et al., 2000). In Japan, approximately 12% of the population expresses a PrP^C^ polymorphism, where the glutamic acid at position 219 is replaced by lysine (K_219_) (Kitamoto and Tateishi, 1994). This polymorphism is associated with protection from sporadic CJD (Shibuya et al., 1998a,b), but not from iatrogenic CJD (dura matter transplant) (Ikawa et al., 2009) or from familial CJD (Furukawa et al., 1995; Seno et al., 2000). Again, these observations support the importance of lysine in the conversion of PrP^C^ to PrP^Sc^. However, once the PrP^Sc^ template is formed, the role of lysines is unclear.

Covalent modification of some amino acids in the PrP^Sc^ template has been associated with a substantial reduction in infectivity. The loss of infectivity was measured using the well-established incubation time bioassay (Prusiner et al., 1980). Diethylpyrocarbonate (DEPC) reacts with histidines to produce the labile ethoxyformyl adduct (McKinley et al., 1981). When highly purified samples from hamsters infected with the Sc237 strain of hamster-adapted scrapie are reacted with DEPC, a reduction of infectivity of 1,000-fold is observed (McKinley et al., 1981). The infectivity lost to DEPC was restored by the reaction with the nucleophile hydroxylamine, which removes an added ethoxyformyl group to restore an intact histidine (McKinley et al., 1981). When DEPC was reacted with the Sc237 PrP^Sc^ in brain homogenates, however, there was no loss of infectivity, presumably due to the endogenous nucleophiles present in those homogenates (Prusiner et al., 1981). Another reagent, β-propiolactone, effectively inactivates viruses, by covalently modifying DNA and to a much lesser extent the lysine in proteins (Uittenbogaard et al., 2011). When it was used to inactivate prions it was partially successful (∼10-fold reduction) (Haig and Clarke, 1968). The extent of the covalent modification of the amino acids present in the PrP^Sc^ template was not determined in either of these experiments. These results indicate that covalent modification of the PrP^Sc^ template has the potential to impede prion replication.

Acetic anhydride (Ac_2_O) and esters of N-hydroxysuccinimide have been used to study the structure of prions (Gong et al., 2011; Silva, 2012; Silva et al., 2016). The extent of this reaction is determined by the chemical environment of a given lysine in the PrP^Sc^ isoform and has been quantitated using Western-blot and mass spectrometry-based analysis (Gong et al., 2011; Silva, 2012; Silva et al., 2016). While these experiments demonstrated methods to quantitate covalent differences in the PrP^Sc^ template, there was no attempt to relate a loss of infectivity to the extent of the covalent modifications of PrP^Sc^.

Prion replication is a template driven process (Wille and Requena, 2018; Spagnolli et al., 2019), where covalent differences between the PrP^Sc^ template and PrP^C^ may influence prion propagation (Supattapone et al., 1999). In principle, covalent modification of lysines in the PrP^Sc^ template may impede its replication, as has been observed when histidines were covalently modified (McKinley et al., 1981). Once the template, containing acetylated lysines, converts PrP^C^ into the prion isoform, the newly formed terminal portion of the template no longer contains acetylated lysines and future prion replication can continue unimpeded from this template with a new terminus. Since prion replication is a template driven and not an enzymatically driven process, the degree of covalent modification of an amino acid essential in prion replication directly determines the extent of the impediment to prion replication. The extent of this impediment can be quantitated by using bioassay to measure the survival time and relate it to an equivalent dilution (Prusiner et al., 1982). The difference between the calculated dilution and the unreacted starting material corresponds to the loss of template due to the acetylation of lysines in that template.

A combined mass spectrometry and antibody-based method was used to quantitate the extent to which the lysines present in the PrP^Sc^ template reacted with acetic anhydride or the acetyl ester of N-hydroxysuccinimide. In addition, rodent bioassay was used to quantify the effects of lysine acetylation on infectivity. These approaches provide insight into surface exposure of the lysines of the wild type Sc237 prion and their importance in prion replication. The results of this analysis are reported below.

## Experimental Section

HPLC grade water was purchased from Burdick and Jackson (Honeywell Research Chemicals; Charlotte, NC, United States). Acetonitrile, HPLC grade, was purchased from Fisher Scientific (Waltham, MA, United States). Trypsin (porcine, sequencing grade, modified) was purchased from Promega (Madison, WI, United States). Chymotrypsin was purchased from Worthington Biochemical Corp. (Lakewood, NJ, United States). All other reagents were purchased from Sigma-Aldrich (St. Louis, MO, United States).

### Production of Recombinant PrP

Recombinant PrP was obtained from plasmids expressing the hamster protein sequence [equivalent to the mature protein sequence (23-231) with an N-terminal methionine]. The plasmids were cloned into BL21 cells (Millipore Sigma; Burlington, MA, United States) and induced to over express hamster recombinant PrP by the addition of isopropyl β-D-1-thiogalactopyranoside (IPTG). The cells were pelleted and the inclusion bodies isolated from that pellet using standard molecular biology techniques (Sambrook et al., 1989).

### Isolation of Recombinant PrP

The inclusion body pellet was suspended in 1 ml of denaturing buffer (6M GuCl, 100 mM sodium phosphate, 10 mM Tris, pH 8.0) and sonicated for 3 min. After sonication the suspension was centrifuged at 20,000 × *g* for 5 min to remove any insoluble material. The supernatant was applied to a 1 ml HIS-Select cartridge (Sigma, St. Louis, MO, United States) that had previously been stripped of nickel (II) ions and recharged with copper (II) ions according to the manufacturer’s instructions. The denatured recombinant protein bound to the cartridge was renatured by the application of a 5-h linear gradient (0.04 ml/min) starting with 100% denaturing buffer and ending with 100% refolding buffer (100 mM sodium phosphate, 10 mM Tris, pH 8.0). After the gradient was completed the cartridge was washed for a further hour with refolding buffer at a flow rate of 0.05 ml/min. The refolded protein was eluted with 5 ml of 0.1 M EDTA and immediately dialyzed against 1L of 100 mM ammonium acetate (pH 4.5) overnight at room temperature, using a dialysis cassette (7000 MWCO; Thermo Fisher Scientific/Pierce). The next day the dialysis buffer was discarded and replaced with 1 L of fresh buffer and allowed to dialyze for an additional 2 h. Aliquots were lyophilized and quantitated by protein assay (Thermo Fisher Scientific/Pierce BCA) and UV/visible spectrophotometry (absorbance at 280 nm). All of the proteins contained an N-terminal methionine (Flinta et al., 1986). The molecular weight of the proteins predicted by the *Prnp* gene sequences matched that observed by mass spectrometric analysis.

The purified ^15^N-labeled Syrian hamster rPrP (^15^NSHarPrP) was analyzed by mass spectrometry. The mass spectrometry-based analysis revealed that the protein was uniformly labeled with ^15^N (>99.7) and could be used, after digestion with trypsin or chymotrypsin, to produce the required internal standards. The analysis also indicated that it contained the expected N-terminal methionine (Flinta et al., 1986).

### Reaction of Recombinant PrP With Ac_2_O or Ac-NHS

Purified natural abundance rPrP was dissolved in one milliliter of reaction buffer (2% w/v octyl β-D-glucopyranoside; 50 mM sodium phosphate, pH 8.0), mixed and then centrifuged (20,000 × *g*; 20 min) to remove any aggregates. 90 μl aliquots of the supernatant were placed into ten microfuge tubes. A 10-μl aliquot of a 0, 1, 10, 50, or 100 mM solution of Ac-NHS or 0, 50, 100, 200, or 500 mM solution of Ac_2_O was added to different tubes. The tubes were rotated at room temperature (21°C) for 15 min. After 15 min, 10 μl of a 1 M Tris solution (pH 8.0) was added, and the tubes were rotated for an additional 15 min at room temperature to quench the reaction. 900 μl of cold (−20°C) methanol was added, mixed, and stored in a −20°C freezer, overnight. The tubes were removed from the freezer and centrifuged (−11°C; 20,000 × *g*; 20 min). The supernatant was removed; the pellet resuspended in 500 μl of cold (−20°C) 85% methanol and centrifuged (−11°C; 20,000 × *g*; 20 min). The supernatant was removed, and the pellet was processed for mass spectrometry-based analysis.

### Animal Handling

Animal experiments were carried out in accordance with the recommendations contained in the Guide for the Care and Use of Laboratory Animals of the National Institutes of Health. The procedures were governed by a protocol that was approved by the Institutional Animal Care and Use Committee of the United States Department of Agriculture, Agricultural Research Service, Albany, CA, United States (Protocol Number: P-10-3). All inoculations and euthanizations were performed under isoflurane anesthesia. LVG Syrian golden hamsters (*Mesocricetus auratus*) were obtained from a commercial vendor (Charles River Laboratories; Wilmington, MA, United States). The Sc237 strain of hamster-adapted scrapie was purchased from InPro Biotechnology (South San Francisco, CA, United States) and passaged once through LVG Syrian golden hamsters.

To minimize adverse impacts of the chemical reagents upon the hamsters, all Ac_2_O and Ac-NHS reaction mixtures containing the Ac_2_O or Ac-NHS reagents were diluted 1:10 with PBS prior to inoculation. No inoculated animal was observed to suffer any short-term ill effects from these inoculations. Each female LVG hamster (4 weeks old), was inoculated intracranially (*ic*) with 50 μl of either one of the brain homogenate (BH) dilutions, one of the dilutions of purified prions, or one of the ten diluted (1:10) reaction mixtures. Six hamsters were inoculated with 50 μl replicates of each sample. Eighty-four animals were inoculated in total. Details on the various inocula can be found in the following methods sections: *Ac-NHS reactions*, *Acetic anhydride (Ac_2_O) reactions*, *Quantitation of loss of infectivity by bioassay*, and *Bioassay of Ac-NHS and Ac_2_O reaction mixtures*. The animals were observed for clinical signs and were humanely euthanized when they were no longer able to feed or drink. The appearance of clinical signs and the date of humane euthanization were recorded.

### Preparation of Samples

Brains were removed from euthanized hamsters in the terminal stages of a prion infection following inoculation (*ic*) with the Sc237 strain of hamster-adapted scrapie. A 10% homogenate (w/v) was prepared from these brains in sucrose buffer (0.32M sucrose in PBS) using an Omni GLH general laboratory homogenizer and disposable Omni Tip plastic generator probes (Omni International; Kennesaw, GA, United States). The homogenate was then centrifuged for 15 min (3,000 × *g*; 20°C), in an Eppendorf 5810R refrigerated centrifuge (Eppendorf; Hamburg, Germany), to remove large particles. The supernatant was retained as the brain homogenate (BH). Some of this homogenate was used as the substrate for the acetic anhydride (Ac_2_O) reaction (*vide infra*) and some was used to obtain purified prions.

PrP^Sc^ was purified according to the methods of Bolton et al. (1991), with some minor modifications (Silva et al., 2011). Briefly, the brain homogenate was diluted with an equivalent volume of 20% w/v N-Lauroylsarcosine sodium salt and 19 mM sodium phosphate, pH 8.5, to yield two volumes of Sarkosyl homogenate. This was allowed to stand for 30 min at room temperature and then centrifuged for 18 min (16,000 × *g*; 20°C), in an Eppendorf 5810R refrigerated centrifuge, to yield the clarified Sarkosyl homogenate. A portion of this clarified homogenate was diluted to 3 ml with buffer (10% w/v N-Lauroylsarcosine sodium salt, 9.5 mM sodium phosphate, pH 8.5) and transferred to an ultracentrifuge tube (4.2 ml, 16 mm × 38 mm). The contents of the tube were underlaid with 1 ml of 20% w/v sucrose and sealed. The sample in the sealed tube was centrifuged for 75 min at 150,000 × *g* (46,000 rpm, 20°C) with a floating Noryl spacer in a Beckman 70.1 Ti rotor (Beckman Coulter; Brea, CA, United States) to obtain the pellet of purified PrP^Sc^. The supernatant was carefully removed and discarded. The pellet was resuspended in 200 μl of buffer (2% w/v octyl β-D-glucopyranoside, 50 mM phosphate buffer, pH 8.0) by brief sonication using closed tubes in a cup horn (four 45-s bursts; Misonix 3000 sonicator; Misonix; Farmingdale, NY, United States). The sonicated suspension of purified PrP^Sc^ was used as a substrate for the N-hydroxysuccinimide ester of acetic acid (Ac-NHS) reaction (*vide infra*).

### β-Propiolactone Reactions

A 10% brain homogenate was prepared by homogenizing a brain (1 g) from a healthy uninfected hamster in 100 mM phosphate buffer (pH 8.0) containing 2% (w/v) octyl β-D-glucopyranoside. After homogenization the solution was centrifuged (20,000 × *g* for 15 min) to remove the suspended particles and yield the clarified brain homogenate. The pellet was discarded. The supernatant was retained and used for the listed reactions.

Nine aliquots of the clarified brain homogenate were apportioned into separate screw-cap microcentrifuge tubes. 10 μl of either a 0, 1, 10, 50, 100, 250, 500, or 1000 mM solution of β-propiolactone in DMSO or a 200 mM solution of Ac-NHS in DMSO was added to one of the 90 μl aliquots. The tubes were rotated at room temperature (21−22°C) for 15 min. After 15 min of reaction, 10 μl of a 1M solution of Tris (pH 8.0) was added to quench the reaction and rotated for an additional 15 min. The samples were precipitated with cold (−20°C) methanol. The pellet was isolated by centrifugation (20,000 × *g*; 20 min; −11°C). 100 μl of Laemmli buffer was added to the dried pellet and boiled for 10 min. A 10-μl aliquot of cooled sample was analyzed by Western blot (probed with 3F4 mAb).

### Ac-NHS Reactions

Twenty individual samples of purified prions were prepared and combined. The combined suspension was aliquoted (180 μl) into twenty 1.5 ml screw cap microcentrifuge tubes. To eight of the forty tubes was added 20 μl of one of the following five concentrations of Ac-NHS in DMSO: 0, 10, 50, 100, or 200 mM. These were rotated for 15 min at room temperature. To quench the reaction, 20 μl of 1 M Tris pH 8.0 was added and the samples were again rotated for 15 min at room temperature.

From each Ac-NHS reaction, three aliquots (110 μl) were retained for mass spectrometry-based analysis, three aliquots were retained for Western blot-based analysis, and one aliquot of each set of Ac-NHS reactions was diluted 10x with PBS before inoculation (*ic*) into hamsters (see section “Bioassay of Ac-NHS and Ac_2_O Reaction Mixtures” below). An aliquot (110 μl) of the unreacted starting material (0 mM Ac-NHS) was further diluted (10^–2^ and 10^–4^) and used to prepare the bioassay calibration curve (see section “Quantitation of Loss of Infectivity by Bioassay” below).

### Acetic Anhydride (Ac_2_O) Reactions

A total of 3.2 ml of the BH were brought up to 4 ml of a solution of 2% octyl β-D-glucopyranoside, 50 mM sodium phosphate pH 8.0, solubilized for 10 min, and then centrifuged for 10 min, 20,000 × *g*, to remove large particles. The supernatant was removed to a new tube and aliquoted (180 μl) into twenty 1.5 ml screw cap microcentrifuge tubes. To each was added 20 μl of one of the following five concentrations of Ac_2_O in DMSO: 0, 50, 100, 200, or 500 mM. These were rotated for 15 min at room temperature. To quench the reaction, 20 μl of 1 M Tris pH 8.0 was added and the samples were again rotated for 15 min at room temperature.

From each Ac_2_O reaction, three aliquots (110 μl) were retained for mass spectrometry-based analysis (*vide infra*), three were retained for Western blot analysis (*vide infra*), and one aliquot was diluted 10x with PBS before inoculation (*ic*) into hamsters (*vide supra*) (see section “Bioassay of Ac-NHS and Ac_2_O Reaction Mixtures” below). One aliquot (110 μl) of the unreacted starting material (0 mM Ac-NHS) was further diluted (10^–2^ and 10^–4^) and used to prepare the bioassay calibration curve (see section “Quantitation of Loss of Infectivity by Bioassay” below).

### Reduction, Alkylation, and Tryptic Cleavage of PrP Samples

The reaction mixtures were inactivated by addition of enough 8M guanidine hydrochloride (GuCl) to achieve a final concentration of 6 M. The solutions stood for 24 h at room temperature. The denatured proteins were precipitated with ice-cold methanol (85% methanol plus 15% protein solution) and centrifuged for 20 min at 20,000 × *g* for 20 min in a cold rotor (−11°C) with an Eppendorf Model 5417R centrifuge (Eppendorf; Hamburg, Germany). The pellets resulting from the methanol precipitation were dissolved in a 10 μl solution (0.01% octyl β-D-glucopyranoside, 1 pmol/μl methionine, and 8% acetonitrile) and sonicated for 5 min. After sonication (Cole-Parmer model 8892; Vernon Hills, IL, United States), a 10 μl solution containing the appropriate internal standard (18 fmol/μl in 0.01% octyl β-D-glucopyranoside, 1 pmol/μl methionine, and 8% acetonitrile) was added. The 20 μl solution was sonicated for an additional 5 min and then 10 μl of a solution of 10 mM dithiothreitol (DTT) in buffer A (25 mM ammonium bicarbonate, 0.01% octyl β-D-glucopyranoside, 1 pmol/μl methionine, and 8% acetonitrile; pH 8.0) was added to the mixture and allowed to react for 1 h at 37°C. After the reaction mixture cooled to room temperature, 40 μl of a solution of 10 mM iodoacetamide in buffer A was added to the mixture and allowed to stand in the dark at room temperature for 1h. The excess iodoacetamide was quenched by the addition of DTT (20 μl of 10 mM DTT in buffer A). The reduced and alkylated proteins were subjected to proteolysis with added trypsin (1 μg trypsin/10 μl water) or chymotrypsin (500 ng chymotrypsin/10 μl of a 20 mM CaCl_2_ solution). The trypsin digestion proceeded at 37°C for 16 h. The chymotrypsin reaction proceeded at 30°C for 18 h, followed by the addition of 2.5 μl of 10% formic acid to stop the reaction. Samples were filtered through 10,000 molecular weight cutoff filters for 12 min at 14,000 × *g*. Samples were stored at −20°C until analyzed.

### Quantitative Mass Spectrometry: Nanospray LCMSMS

An Applied Biosystems (Sciex; Framingham, MA, United States) Model 4000 Q-Trap instrument equipped with a nano-electrospray source was used to perform NanoLC-MS/MS. 6 ml aliquots of each digest (10.8 fmol internal standard) were loaded onto a C-18 trapping cartridge (PepMap, 5 μm, 100A, 300 μm i.d. × 5 mm, flow rate 15 μl/min; Thermo Fisher Scientific/Dionex; Sunnyvale, CA, United States). Salts were washed from the cartridge with a solution of acetic acid/acetonitrile/heptafluorobutyric acid/water (0.5/1/0.02/99). The salt-free bound peptides were eluted onto a reverse-phase column (Vydac Everest 238EV5.07515, 75 μm × 150 mm, flow rate 250 nL/min, Hichrom; Leicestershire, United Kingdom).

The solvents were delivered with an Applied Biosystems model Tempo nanoflow LC system (Sciex) with autosampler, column switching device, and nanoflow solvent delivery system. Samples were eluted from the column with a binary gradient (A, 0.5% acetic acid in water and B, 80% acetonitrile, 0.5% acetic acid). The flow rate was 250 nl/min with a 16-min linear gradient starting with 5% B and ending with 100% B. Elution with 100% B was held for 7 min followed by a return to 5% B over 4 min. The eluted samples were sprayed with non-coated spray tip (FS360-20-10-N-20-C12, New Objective Inc., Woburn, MA, United States) onto the Applied Biosystems source, Model Nanospray II.

The instrument response for the peptides **NKP**SKPKTNM (NKP), **PGG**WNTGGSR (PGG), and **QHT**VTTTTK (QHT) was optimized by a previously described method (Onisko et al., 2007). Briefly, a recombinant PrP digest (*vide supra*) or synthetic peptide (1 pmol/μl; 20 μl) dissolved in solvent (50/49.5/0.5; acetonitrile/water/acetic acid) was isocratically eluted from the reverse-phase column at a flow rate of 250 nL/min. The analyte signal was maximized by adjusting the source parameters (electrospray voltage, curtain gas and nebulizing gas settings, ion source heater temperature, declustering potential (DP), and nanospray tip positioning relative to the front plate orifice). The intensity of specified product ions was optimized by adjusting the Q2 offset voltage [“collision energy” (CE)] to yield optimal fragmentation. The mass settings for the three peptides are the following: NKPSKPKTNM [precursor ion *m*/*z* (*z* = 3) of 382.2, product ion of 226.1 (b_2_-NH_3_) or 590.3 (y_5_)], PGGWNTGGSR [precursor ion *m*/*z* (*z* = 2) of 494.8, product ion of 891.5 (y_9_)], and QHTVTTTTK [precursor ion *m*/*z* (*z* = 2) of 509.1, product ion of 751.4 (y_7_)]. The optimal CEs for the peptides were determined to be 30 V for ions from NKPSKPKTNM and PGGWNTGGSR and 26 V for the ion from QHTVTTTTK. The optimal DPs for the b_2_-NH_3_ and y_5_ ions from the peptide NKPSKPKTNM, the y9 ion from the peptide PGGWNTGGSR, and the y7 ion from the peptide QHTVTTTTK were determined to be 80, 90, 110, and 110 V, respectively. The mass spectrometer was operated in multiple reaction monitoring (MRM) mode, alternating between detection of the desired peptides and the appropriate internal standards.

The **NKP**SKPKTNM (NKP) peptide contains a methionine which can be oxidized (Silva et al., 2010, 2011, 2013). We determined that the peptide and its oxidized homolog are chromatographically separable. We employed our previously described method to quantitate the portion of the oxidized peptide (Silva et al., 2010, 2011, 2013). We determined that the oxidized peptide was found to be present in all of the samples and that the amount was consistently low (<5%). Therefore, we did not include it in any of our calculations. The peptide YRPVDQY does not contain a lysine and is unaffected by reaction with Ac_2_O or Ac-NHS, so it was used to normalize samples digested with chymotrypsin.

### Mass Spectrometry-Based Quantitation of Tryptic and Chymotryptic Peptides

Hamster PrP was digested with either trypsin or chymotrypsin using previously described conditions (Onisko et al., 2007). Trypsin digestion yields the lysine associated peptides **PGG**WNTGGSR (PGG), **QHT**VTTTTK (QHT), **GEN**FTETDIK (GEN), **VVE**QMCTTQYQK (VVE) and **ESQ**AYYDGR (ESQ). The optimized instrument response for the YPGQGSPGGNR, GEN, VVE, and ESQ peptides has been previously reported (Douma et al., 1998; Onisko et al., 2007; Silva et al., 2011). Chymotryptic digestion requires overnight digestion (30°C) for optimal results and yields the peptides **NKP**SKPKTNM (NKP) and YRPVDQY. The optimized instrument parameters for the peptide YRPVDQY have been previously reported (Sturm et al., 2012).

^15^N-labeled rPrP was used as an internal standard. The isotopic purity of the uniformly labeled product was determined to be 99.7%. None of the peptides derived from ^15^N-labeled rPrP interfered with the analysis of any other ^15^N-labeled peptides or with any of the peptides derived from rPrP or PrP^Sc^ samples. The area ratios of the MRM transition signals from the tryptic peptides PGG (1.14 ± 0.03), QHT (0.22 ± 0.01), GEN (1.7 ± 0.07), VVE (0.084 ± 0.004), and ESQ (1.24 ± 0.03) peptides to YPGQGSPGGNR varied by less than 10% (*n* = 180). The area ratio of the MRM transition signals from the chymotryptic NKP (0.23 ± 0.02) to the YRPVDQY peptide also varied by less than 10% (*n* = 180). The YPGQGSPGGNR and YRPVDQY peptides do not contain lysines and are produced in proportion to the progenitor PrP protein, so they were used to normalize the MRM transition signals from the other tryptic or chymotryptic peptides, respectively, to the amount of PrP in the sample after trypsin or chymotrypsin digestion, respectively. Thus, normalizing labile peptide to an inert internal peptide is an effective means of correcting for the differences in the amount of PrP in each sample.

### Western Blot

Protein gels were purchased from a commercial vendor (Thermo Fisher Scientific; Waltham, MA, United States) and used according to the manufacturer’s instructions. The primary antibody 3F4 (Covance; San Diego, CA, United States) and secondary antibody Anti-Mouse IgG (Fc specific)-AP (Sigma; St. Louis, MO, United States) were purchased. Blotting paper, membrane [polyvinylidene fluoride (PVDF)] and SDS−PAGE gels were presoaked in transfer buffer (25 mM Tris, 250 mM glycine in 15% methanol) for 10 min. The proteins were transferred from the gel to the PVDF membrane using a BIO-RAD *Trans*-Blot SD semi-dry transfer cell (50 min at 100 mA). After transfer, the PVDF membrane was placed into a square Petri dish (10 cm × 10 cm) and gently agitated in 20 ml of wash buffer (PBS + 0.05 Tween 20) for 5 min. After 5 min, the wash buffer was discarded, and the membrane was blocked (15 min in 10 ml of StartingBlock T20; Thermo Fisher Scientific/Pierce) with gentle agitation. The membrane was then incubated for 1 h at room temperature in 10 ml of blocking buffer with 1 μl of primary antibody (3F4; 2 mg/mL). After this incubation, the buffer was discarded, and 20 ml of wash buffer was added and gently agitated for 5 min. This was repeated three times with fresh wash buffer. 10 ml of blocking buffer with 10 μl of the secondary antibody was added and incubated at room temperature for 45 min. After incubation, the membrane was washed four times with wash buffer at 5-min intervals. The wash membrane was placed in 10 ml of a Sigma/FAST BCIP/NBT solution (1 tablet per 10 ml per manufacturer’s instructions) and incubated at 37°C. After development, the blots were dried and scanned to obtain densitometry measurements.

### Western Blot-Based Quantitation of Lysine at Position 110 (K_110_)

Five sets of dilutions (undiluted, 1:3.2, 1:10, 1:32, and 1:100) of a PrP 27-30 standard were prepared and each set was analyzed on a separate Western blot. The signal intensity, as measured by densitometry, was determined for the diglycosylated band of PrP 27-30 of each dilution (*n* = 5). The 10 kDa region in each lane was used as background and subtracted from the diglycosylated band in each lane. The relative signal intensity was calculated by normalizing the intensity of the signal (less background) from a dilution to that of the undiluted starting material (less background) for each blot. A calibration curve relating the normalized signal intensities of a dilution to the corresponding log_10_ of its experimental dilution was prepared.

Equivalent amounts of the reaction mixtures for Ac_2_O (0, 5, 10, 20, and 50 mM) with BH and Ac-NHS (0, 1, 5, 10, and 20 mM) with purified prions were analyzed (*n* = 3) on separate Western blots. The calibration curve (*vide supra*) was used to relate the observed normalized signal intensity of the diglycosylated band of each PrP^Sc^ sample (*vide supra*) to a log dilution. This data was used to determine the relative extent of the reaction of K_110_ with Ac_2_O or Ac-NHS.

### Densitometry

Densitometry measurements were made using the AlphaEaseFC version 4.0.0 software (Alpha Innotech/Genetic Technologies, Inc., Miami, FL, United States) with black having a value of 255 and white having a value of 0. The statistical analysis was performed with Microsoft Excel.

### Quantitation of Loss of Infectivity by Bioassay

Dilutions of BH or purified PrP^Sc^ preparations, derived from the brains of prion-infected hamsters, were used to prepare calibration curves relating survival times to their corresponding log_10_ dilutions. Two sets (BH or purified prions) of three dilutions (10^–1^, 10^–3^, or 10^–5^) were inoculated (*ic*) into an animal (10^–3^ and 10^–5^ dilutions of 0 mM Ac-NHS and Ac_2_O × 6 animals per dilution = 24 animals). The survival times were recorded and used to prepare the calibration curves for the BH and the purified preparations.

### Bioassay of Ac-NHS and Ac_2_O Reaction Mixtures

The prions contained in the BH were reacted with one of five final concentrations (0, 5, 10, 20, and 50 mM) of Ac_2_O. Each reaction mixture was diluted 1:10 in phosphate buffered saline (PBS); a 50 μl aliquot of a dilution was injected (*ic*) into a hamster (5 reaction mixtures × 6 animals per reaction mixture = 30 animals). The animals were observed for the presentation of clinical signs of advanced prion disease, then humanely euthanized. The time from the date of the inoculation to the date of humane euthanization, the survival time, was determined for each set of inoculated reaction mixtures. The bioassay calibration curves for the BH were used to relate those observed survival times to their corresponding log_10_ dilutions.

Aliquots of the purified prions were reacted with five different final concentrations (0, 1, 5, 10, or 20 mM) of Ac-NHS. Again, each reaction mixture was diluted 1:10; a 50 μl aliquot of a dilution was injected (ic) into a hamster (5 reaction mixtures × 6 animals per reaction mixture = 30 animals). The observed survival times were recorded and used to calculate the corresponding log_10_ dilutions, based on the purified prion calibration curves.

### Confirming the Presence on Prions in Infected Animals

A portion of the brain (∼50 mg) from two of the six animals in each set was removed and homogenized in 300 μl of PBS. The homogenate was clarified and divided into two portions. One was treated with proteinase K (50 μg/ml; 37°C; 1 h) and the other was not. The prions in the samples were inactivated by the addition of 3 volumes of 8M guanidine hydrochloride (GuCl) (Prusiner et al., 1993). The solutions were mixed and allowed to stand for 24 h at room temperature. The GuCl solutions were individually precipitated with cold (−20°C) aqueous methanol (85% methanol). The resulting pellets were suspended in Laemmli buffer, boiled (10 min) and then analyzed by Western blot (3F4 primary antibody) to confirm the presence of prions in that animal’s brain.

These samples were tested for the presence of prions by Western blot (3F4 primary antibody). The resulting Western blot analysis (data not shown) showed that these samples were indistinguishable from Sc237 controls. In addition, each animal displayed clinical signs that were consistent with being infected with the Sc237 strain of hamster-adapted scrapie.

### Statistical Analysis

The bioassay and the mass spectrometry data were statistically analyzed. The unpaired Student’s *t*-test was used to compare the relative peptide quantitation data from specific chemical reactions to the measured loss of infectivity of inoculated reaction mixtures. It was also used to compare the relative peptide quantitation data from specific chemical reactions to other chemical reactions. Linear least squares regression was used to prepare the bioassay calibration curves and the curves measuring the relationship between the extent of the reaction of individual peptides *v*. the concentration of the reagents. This analysis yielded the slope and intercept of those linear equations. *p*-Values for linear regression were determined using standard statistical methods^1^. The slopes of the regression lines derived from linear regression analysis were compared using a *t*-test^2^. Excel was used to perform all calculations.

### Safety Considerations

All prion-containing samples were manipulated in a dedicated biosafety level 2 (BSL-2) laboratory, which was certified and inspected by the Animal and Plant Health Inspection Service (APHIS^3^) using procedures outlined in the Biosafety in Microbiological and Biomedical Laboratories, fifth edition^4^. The infectious prions were inactivated before removal from the BL2 laboratory (Prusiner et al., 1993). Acetonitrile is hazardous and was manipulated in a dedicated chemical safety hood.

## Results

### Acetic Anhydride (Ac_2_O) and Ac-NHS Were Selected to React With the Lysines of Prion Template

Acetic anhydride, Ac-NHS, diethylpyrocarbonate, and β-propiolactone have been used to covalently modify prions (Haig and Clarke, 1968; Gong et al., 2011; Silva, 2012; Silva et al., 2016). The diethylpyrocarbonate (DEPC) reagent was inhibited by the endogenous nucleophiles present in the brain homogenate (McKinley et al., 1981; Prusiner et al., 1981). β-propiolactone has been used to inactivate viruses and shown to react with lysines (Uittenbogaard et al., 2011). β-Propiolactone was evaluated to determine if its reactivity was influenced by external nucleophiles.

**FIGURE 1:**
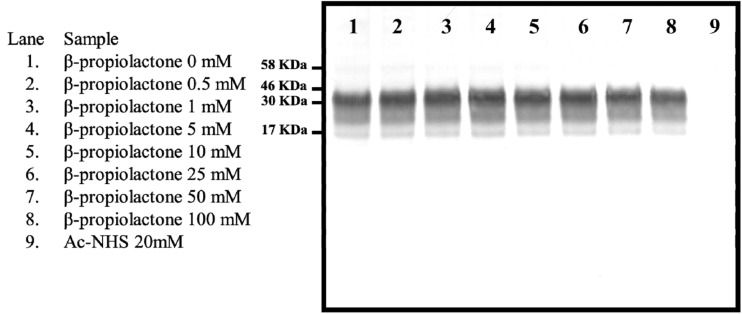
Western blot showing the intensity of the signal from the reaction of various concentrations of β-propiolactone or Ac-NHS (20 mM) with the PrP^C^ present in a 10% brain homogenate from an uninfected hamster. The Western blot was probed with the 3F4 mAb.

β-propiolactone has been previously shown to partially inactivate prions (Haig and Clarke, 1968). We assessed β-propiolactone’s ability to covalently modify PrP^C^’s lysines by measuring the extent of propionation of lysine 110 (K_110_). Each of nine supernatant samples from uninfected hamster brain homogenate were reacted with one of eight different concentrations of β-propiolactone or one concentration of Ac-NHS. The reaction mixtures were analyzed by Western blot, using the 3F4 mAb (Figure 1), and the resulting signals were quantified by densitometry. The signal intensity for each β-propiolactone reaction (Figure 1, lanes 2-8), relative to the unreacted starting material (Figure 1, lane 1), varied by only 6% (1.04 ± 0.06). The signal intensity for the Ac-NHS reaction (Figure 1, lane 9) was the same as background. This indicates that β-propiolactone reacts preferentially with molecules in the brain homogenate other than lysines in PrP^C^ and therefore was not suitable to assess the effect of lysine acetylation on prion inactivation.

### Quantitation of Tryptic and Chymotryptic Peptides Can Be Related to the Acetylation of Lysine at Position 23, 24, 27, 101, 104, 106, 185, 194, 204, or 220 (K_23_, K_24_, K_27_, K_101_, K_104_, K_106_, K_185_, K_194_, K_204_, and K_220_)

**FIGURE 2:**
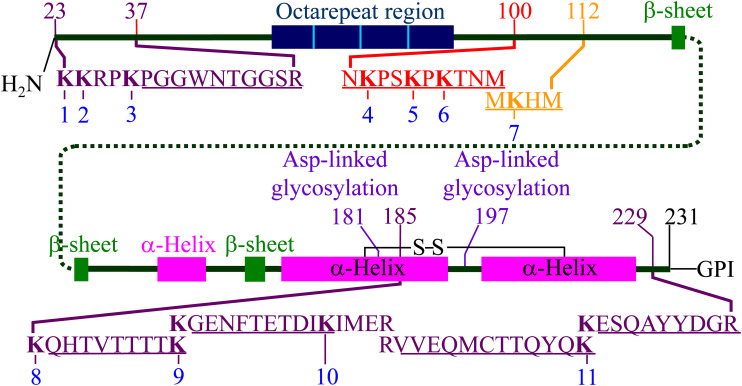
Representation of hamster PrP^C^ including the locations of secondary structures and the position of the 11 lysine residues. The locations of the five tryptic peptides (**PGG**WNTGGSR, **QHT**VTTTTK, **GEN**FTETDIK, **VVE**QMCTTQYQK, and **ESQ**AYYDGR) are indicated. The locations of the chymotryptic peptide (**NKP**SKPKTNM) and the 3F4 mAb epitope (**MKH**M) are also indicated. Adapted from Silva et al. (2016). The locations of the secondary structure motifs are based on the NMR structure (1B10 in PDB) (James et al., 1997).

Acetylation of the ε-amino group of lysine precludes its cleavage by trypsin. Acetylation of K_27_, K_185_, K_194_, or K_220_ which precede the peptide **PGG**WNTGGSR (PGG), **QHT**VTTTTK (QHT), **GEN**FTETDIK (GEN), or **ESQ**AYYDGR (ESQ) would prevent that peptide from being produced by trypsin digestion (Figure 2). Acetylation of K_194_, K_204_, or K_220_ which terminates QHT, GEN, or **VVE**QMCTTQYQK (VVE), would also prevent that peptide from being produced by tryptic cleavage. Therefore, the extent of the acetylation of K_27_, K_185_, K_194_, K_204_, or K_220_ can be quantitated by determining the amount of each peptide present after trypsin digestion relative to its abundance in a corresponding trypsin digest of unreacted starting material. K_101_, K_104_, and K_106_ are contained in the chymotryptic **NKP**SKPKTNM (NKP) peptide; acetylation of any one of these lysines results in the peptide being undetectable by our mass spectrometry-based method. Thus, a mass spectrometry-based analysis can be used to relate the extent of acetylation of a specific lysine to the loss of signal from a defined tryptic or chymotryptic peptide.

### Western Blot Was Used to Quantitate the Acetylation of K_110_

**FIGURE 3:**
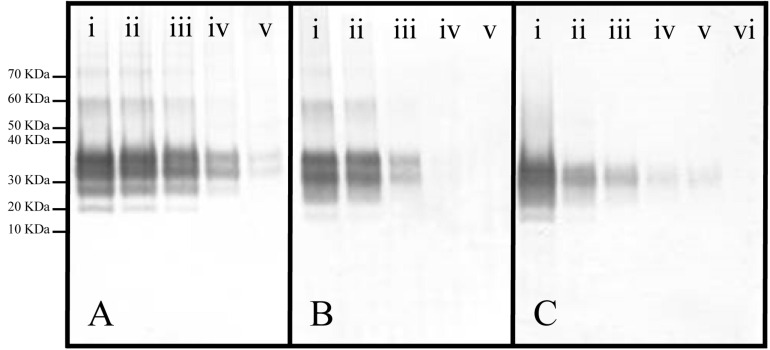
Representative Western blots of panels **(A)** Ac_2_O or **(B)** Ac-NHS reaction mixtures and **(C)** a dilution series of PrP 27-30 (primary mAb, 3F4). **(A)** Equal aliquots of a brain homogenate (BH) were reacted with either 0 (i), 5 (ii), 10 (iii), 20 (iv), or 50 mM (v) Ac_2_O (*vide supra*). **(B)** Aliquots of purified PrP^Sc^ were reacted with either 0 (i), 1 (ii), 5 (iii), 10 (iv), or 20 (v) mM Ac-NHS. **(C)** PrP 27-30 was undiluted (i) or diluted 1:3.2 (ii), 1:10 (iii), 1:32 (iv), 1:100 (v), or 1:320 (vi).

**FIGURE 4:**
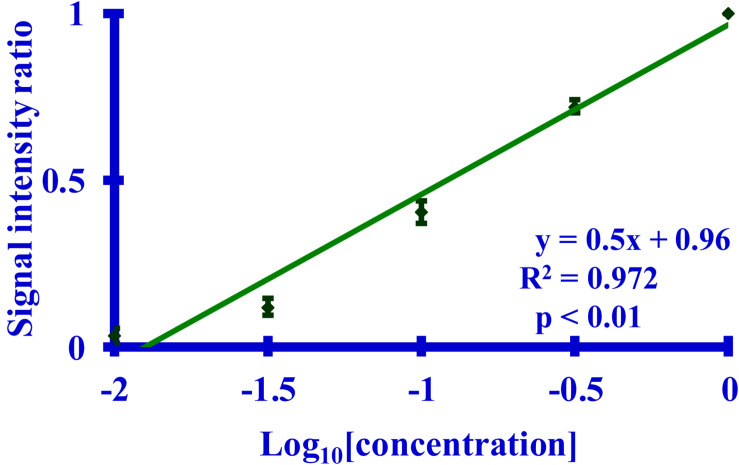
A graph of a Western blot-based relationship between the calculated signal intensity ratio derived from the binding of the 3F4 mAb to its **MKH**M epitope and the corresponding dilution of PrP 27-30. The primary mAb was 3F4. The calculated signal intensity is the ratio of the signal from a dilution to the undiluted starting material, as measured by densitometry. Five dilutions (*n* = 5 per dilution) of PrP 27-30 (undiluted, 1:3.2, 1:10, 1:32, or 1:100) were used.

A Western blot, probed with the 3F4 antibody, was be used to quantitate the extent of acetylation of K_110_ (Figure 3). A calibration curve relating the relative signal intensity from a Western blot to the corresponding dilution was prepared. The resulting curve (Figure 4) was linear with an excellent correlation coefficient (R^2^ = 0.972, *p* < 0.01). This curve was used to calculate the log_10_ dilution corresponding to the ratio of the observed signal intensity to that of unreacted starting material for each reaction set.

### The Lysines of rPrP React Equally Well With Either Ac_2_O or Ac-NHS

**FIGURE 5:**
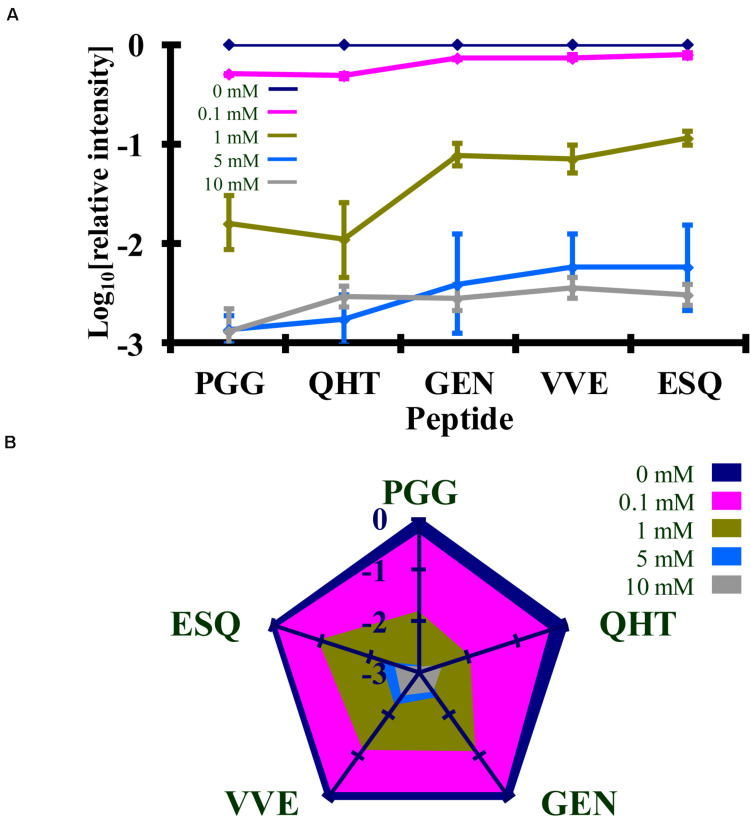
Comparison of the changes in the log_10_ of the normalized MRM transition signals from five tryptic peptides (**PGG**WNTGGSR, **QHT**VTTTTK, **GEN**FTETDIK, **VVE**QMCTTQYQK, and **ESQ**AYYDGR) after the reaction of rPrP with Ac_2_O. **(A)** Graphical representation of the data. **(B)** Radar plots of this data. Each rPrP reaction was performed in duplicate. The locations of the peptides may be found in Figure 2.

**FIGURE 6:**
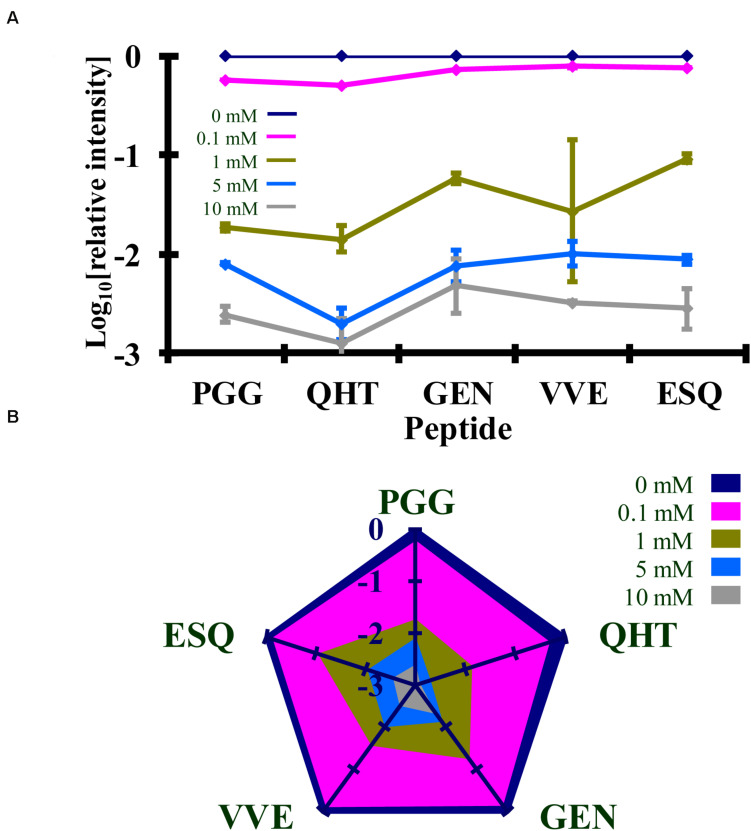
Comparison of the changes in the log_10_ of the normalized MRM transition signals from five tryptic peptides (**PGG**WNTGGSR, **QHT**VTTTTK, **GEN**FTETDIK, **VVE**QMCTTQYQK, and **ESQ**AYYDGR) after the reaction of rPrP with Ac-NHS. **(A)** Graphical representation of the data. **(B)** Radar plots of this data. Each rPrP reaction was performed in duplicate. The locations of the peptides may be found in Figure 2.

Syrian hamster rPrP was reacted with five concentrations of Ac_2_O or Ac-NHS. The reduction of the normalized MRM transition signal (log_10_) of these peptides corresponds to the extent of lysine acetylation at positions 23, 24, 27, 185, 194, 204, and 220 (K_23_, K_24_, K_27_, K_185_, K_194_, K_204_, and K_220_). The log_10_ of the normalized MRM transition signal for each of the five peptides after reaction with each concentration of Ac_2_O or Ac-NHS was determined (Figures 5, 6). Linear regression was used to calculate the slope, intercept, R^2^, and *p*-value for the relationship between reagent concentration and the normalized MRM transition signal intensity (log_10_) for each peptide. The data is summarized in Tables 1A, 2A. The slopes from the regression were statistically compared and no significant differences were observed between the slopes for any of the peptides reacted with Ac_2_O (*p* > 0.66) or Ac-NHS (*p* > 0.57) (Tables 1B, 2B). No differences (*p* > 0.62) were observed when the slope of each peptide reacted with Ac_2_O was compared to the slope of same peptide reacted with Ac-NHS (Table 3A). No significant difference (*p* > 0.25) was observed between the reaction of a given lysine with either Ac_2_O or Ac-NHS at the 1, 5, or 10 mM concentrations (Table 3B). These results indicate that at the same concentration, both Ac_2_O and Ac-NHS react indiscriminately with the lysines in rPrP.

**TABLE 1:** Summary of statistical data for the reaction of rPrP with Ac_2_O.

**A**						
	**Ac_2_0**	**PGG**	**QHT**	**GEN**	**WE**	**ESQ**
	Slope	−0.3	−0.2	−0.3	−0.2	−0.3
	Intercept	−0.7	−0.8	−0.4	−0.4	−0.4
	R^2^ *p*-value	0.691	0.569	0.785	0.792	0.832
		*p* < 0.01	*p* < 0.05	*p* < 0.01	*p* < 0.01	*p* < 0.01
**B**						
	***t*-test**	**PGG**	**QHT**	**GEN**	**WE**	**ESQ**

	PGG	—	*p* > 0.66	*p* > 0.84	*p* > 0.69	*p* > 0.82
	QHT		—	*p* > 0.76	*p* > 0.88	*p* > 0.75
	GEN			—	*p* > 0.82	*p* > 0.99
	VVE				—	*p* > 0.82
	ESQ					—

***(A)** Table of regression data (slope, intercept, R^2^, *p*-value) for normalized MRM transition signals (log_10_) of each peptide *v*. the Ac_2_O concentration. **(B)** Summary of *t*-tests comparing the slopes of the linear regression of each peptide *v*. a different peptide. p < 0.05 indicate statistical significance. Each reaction was performed on PrP in duplicate. The locations of the peptides may be found in Figure 2.*

**TABLE 2:** Summary of statistical data for the reaction of rPrP with Ac-NHS.

**A**					
**Ac-NHS**	**PGG**	**QHT**	**GEN**	**WE**	**ESQ**
Slope	−0.2	−0.3	−0.2	−0.2	−0.2
Intercept	−0.6	−0.7	−0.5	−0.5	−0.4
R^2^	0.714	0.702	0.762	0.699	0.867
*p*-value	*p* < 0.01	*p* < 0.01	*p* < 0.01	*p* < 0.01	*p* < 0.01
**B**					
***t*-test**	**PGG**	**QHT**	**GEN**	**WE**	**ESQ**

PGG	−	*p* > 0.65	*p* > 0.92	*p* > 0.96	*p* > 0.75
QHT		−	*p* > 0.57	*p* > 0.63	*p* > 0.81
GEN			−	*p* > 0.95	*p* > 0.64
VVE				−	*p* > 0.72
ESQ					−

***(A)** Table of regression data (slope, intercept, R^2^, *p*-value) for normalized MRM transition signals (log_10_) of each peptide *v*. the Ac-NHS concentration. **(B)** Summary of *t*-tests comparing the slopes of the linear regression of each peptide *v*. a different peptide. p < 0.05 indicate statistical significance. Each reaction was performed on PrP in duplicate. The locations of the peptides may be found in Figure 2.*

**TABLE 3:** Statistical analysis of the comparison of the changes in the MRM transition signals (log_10_) from five tryptic peptides (**PGG**WNTGGSR, **QHT**VTTTTK, **GEN**FTETDIK, **VVE**QMCTTQYQK, and **ESQ**AYYDGR) after the reaction of Ac_2_O with rPrP.

**A**						
	***t*-test**	**PGG**	**QHT**	**GEN**	**VVE**	**ESQ**
	*p*-value	*p* > 0.62	*p* > 0.69	*p* > 0.63	*p* > 0.85	*p* > 0.94
**B**						
	***t*-test**	**PGG**	**QHT**	**GEN**	**VVE**	**ESQ**

	0.1 mM	p < 0.02	*p* > 0.46	*p* > 0.85	*p* > 0.51	*p* > 0.61
	1 mM	*p* > 0.95	*p* > 0.98	*p* > 0.87	*p* > 0.70	*p* > 0.89
	5 mM	*p* > 0.79	*p* > 0.76	*p* > 0.33	*p* > 0.56	*p* > 0.27
	10 mM	*p* > 0.41	*p* > 0.74	*p* > 0.97	*p* > 0.83	*p* > 0.94

***(A)** The slopes of the normalized MRM transition signals (log_10_) of each peptide after reaction with five concentrations of Ac_2_O *v*. the same concentrations of Ac-NHS were compared by a *t*-test. **(B)** Unpaired Student’s *t*-test comparing the normalized MRM transition signal (log_10_) of each peptide reacted with the same concentration of Ac_2_O or Ac-NHS. p < 0.05 indicate statistical significance. The locations of the peptides may be found in Figure 2.*

### Calibration Curves From the Bioassay

**FIGURE 7:**
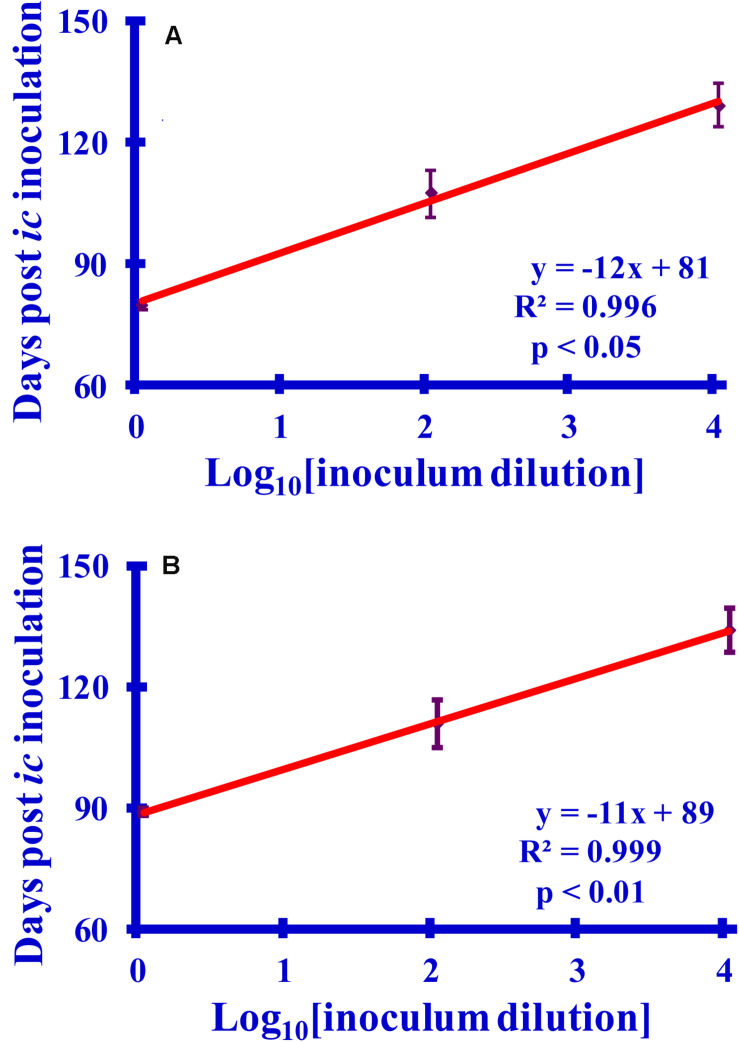
Graphs relating the survival times to log_10_ dilutions (10×, 1000×, or 100,000×) of **(A)** purified PrP^Sc^ or **(B)** PrP^Sc^-infected brain homogenate (BH). The survival times (6 hamsters per set) are recorded as days post-inoculation. For consistency with the reactions, which are diluted 1:10 prior to inoculation, the dilutions are reported relative to a 1:10 dilution of the starting sample.

The observed incubation time of a prion disease can be related to the concentration of the prion inoculum (Prusiner et al., 1982). Calibration curves relating hamster incubation times to dilutions of prion-containing brain homogenate (BH) or purified prions were prepared. The calibration curves relating the survival times to the dilution of infectivity are linear with excellent correlation coefficients (R^2^ > 0.995, *p* < 0.05) (Figure 7). The animals injected with purified preparations have noticeably shorter survival times than do those inoculated with comparable dilutions of BH, which is an expected consequence of using purified prions (Sajnani et al., 2012). These curves allow the calculation of the reduction of infectivity associated with lysine acetylation in BH or purified preparations from the observed survival times.

### Comparing Lysine Acetylation in PrP^Sc^ After Reaction With Ac_2_O or Ac-NHS

**FIGURE 8:**
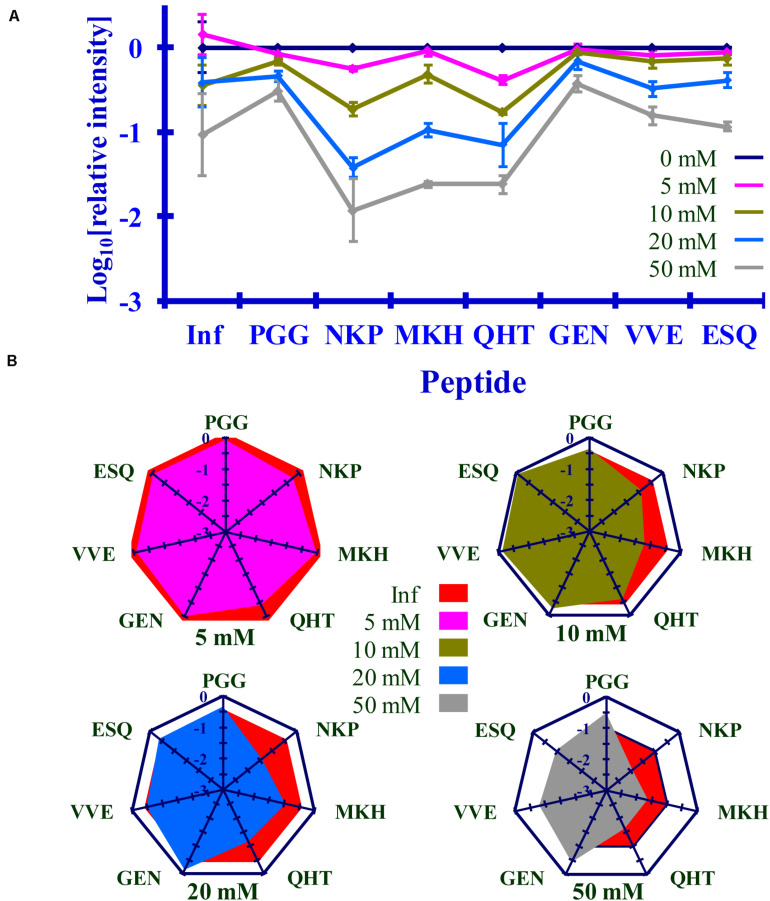
Comparison of the changes in infectivity (Inf) (log_10_) to the normalized MRM transition signals (log_10_) of five tryptic peptides (**PGG**WNTGGSR, **QHT**VTTTTK, **GEN**FTETDIK, **VVE**QMCTTQYQK, and **ESQ**AYYDGR), one chymotryptic peptide (**NKP**SKPKTNM), and the 3F4 epitope (**MKH**M) after the reaction of Ac_2_O with PrP^Sc^-infected brain homogenate. **(A)** Graphical summary of this data. **(B)** Radar plots of this data. Each PrP^Sc^ reaction was performed in triplicate. Hamster bioassay (*n* = 6 per reagent concentration) was used to measure the infectivity. The locations of the peptides may be found in Figure 2.

**FIGURE 9:**
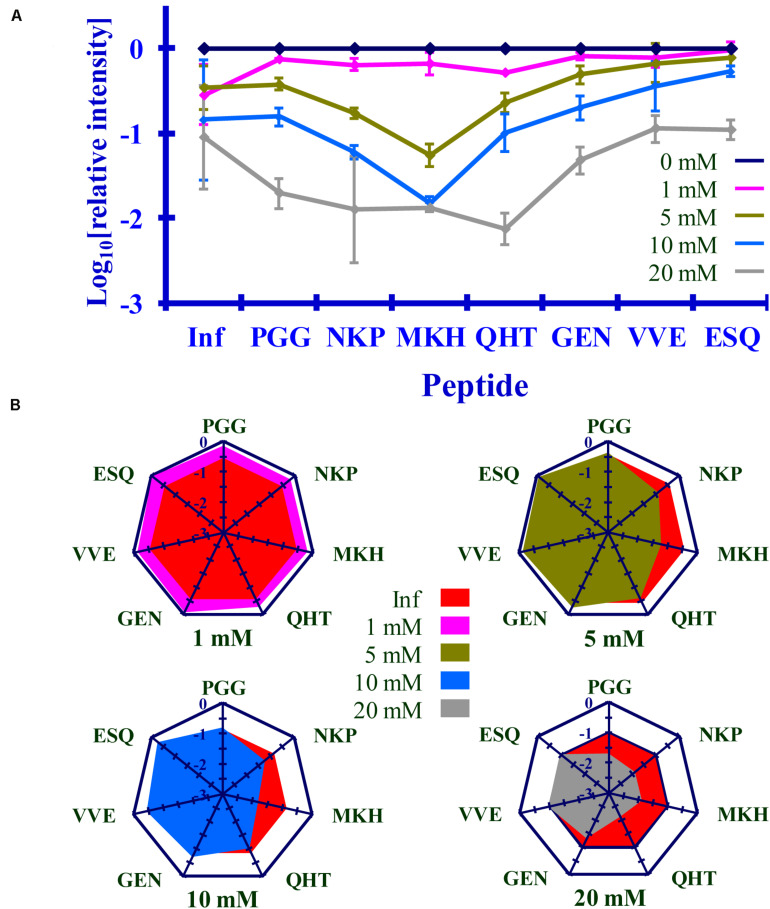
Comparison of the changes in infectivity (Inf) (log_10_) to the normalized MRM transition signals (log_10_) of five tryptic peptides (PGGWNTGGSR, QHTVTTTTK, GENFTETDIK, VVEQMCTTQYQK, and ESQAYYDGR), one chymotryptic peptide (NKPSKPKTNM), and the 3F4 epitope (MKHM) after the reaction of Ac-NHS with purified PrP^Sc^. **(A)** Graphical representation of this data. **(B)** Radar plots comparing changes in infectivity with the extent of the reaction of each peptide at the four Ac-NHS concentrations (1, 5, 10, or 20 mM). Each PrP^Sc^ reaction was performed in triplicate. Hamster bioassay (*n* = 6 per reagent concentration) was used to measure the infectivity. The locations of the peptides may be found in Figure 2.

The normalized MRM transition signals (log_10_) for the five other tryptic and one chymotryptic peptides were determined for each reaction sample. The log_10_-based reduction of infectivity was derived from the bioassays. These values are reported in Figures 8, 9. Linear regression was used to calculate the slope, intercept, R^2^, and *p*-value for the relationship between reagent concentration and the log_10_ loss of infectivity of the normalized MRM transition signal for each peptide (Tables 4A, 5A). When the slopes of the linear regression of the peptides were statistically compared (*t*-test, Tables 4B, 5B), significant differences were found among the peptides (Tables 4B, 5B). These tables show that PrP^Sc^’s lysines, unlike those of rPrP, react differently, depending on the reagent used and, presumably, their chemical environment (Silva, 2012; Silva et al., 2016).

**TABLE 4:** Summary of statistical data for the reaction of Ac_2_O with PrP^Sc^-infected brain homogenate.

**A**									
	**Ac_2_O**	**Inf**	**PGG**	**NKP**	**MKH**	**QHT**	**GEN**	**VVE**	**ESQ**
	Slope	−0.02	−0.01	−0.04	−0.03	−0.03	−0.01	−0.02	−0.02
	Intercept	0.04	−0.06	−0.23	0	−0.31	0	−0.05	0.01
	*R*^2^	0.923	0.968	0.938	0.963	0.915	0.985	0.973	0.993
	*p*-value	*p* < 0.03	*p* < 0.01	*p* < 0.02	*p* < 0.01	*p* < 0.03	*p* < 0.01	*p* < 0.01	*p* < 0.01
**B**									
	**Ac_2_O**	**Inf**	**PGG**	**NKP**	**MKH**	**QHT**	**GEN**	**VVE**	**ESQ**

	Inf	–	*p* > 0.07	*p* > 0.15	*p* > 0.13	*p* > 0.38	*p* < 0.05	*p* > 0.36	*p* > 0.65
	PGG		–	*p* < 0.02	*p* < 0.01	*p* < 0.03	*p* > 0.58	*p* > 0.05	*p* < 0.01
	NKP			–	*p* > 0.7	*p* > 0.47	*p* < 0.02	*p* < 0.05	*p* > 0.06
	MKH				–	*p* > 0.62	*p* < 0.01	*p* < 0.02	*p* < 0.03
	QHT					–	*p* < 0.03	*p* > 0.1	*p* > 0.17
	GEN						–	*p* < 0.02	*p* < 0.01
	VVE							–	*p* > 0.23
	ESQ								–
**C**									
	**Inf**	**PGG**	**NKP**	**MKH**	**QHT**	**GEN**	**VVE**	**ESQ**	

	5 mM	*p* > 0.05	*p* < 0.01	*p* < 0.05	*p* < 0.01	*p* > 0.15	*p* < 0.05	*p* > 0.05	
	10 mM	*p* < 0.05	*p* < 0.05	*p* < 0.01	*p* < 0.05	*p* < 0.01	*p* < 0.05	*p* < 0.05	
	20 mM	*p* > 0.56	*p* < 0.01	*p* < 0.01	*p* < 0.05	*p* > 0.05	*p* > 0.60	*p* > 0.85	
	50 mM	*p* > 0.10	*p* < 0.05	*p* < 0.01	*p* < 0.05	*p* > 0.05	*p* > 0.60	*p* > 0.94	

***(A)** Table of linear regression data (slope, intercept, *R*^2^, *p*-value) relating the change in infectivity (Inf) or the normalized MRM transition signals (log_10_) of listed peptides for each concentration of Ac_2_O. **(B)***t*-test comparing the slopes of the linear regression from infectivity (Inf) *v*. the normalized MRM transition signal (log_10_) of each listed peptide and the normalized MRM transition signal (log_10_) of each peptide *v*. another peptide. **(C)** Unpaired Student’s *t*-test comparison of the infectivity to the normalized MRM transition signal (log_10_) of a peptide at a specific reagent concentration. p < 0.05 indicate statistical significance. Each PrP^Sc^ reaction was performed in triplicate. Hamster bioassay (*n* = 6 per reagent concentration) was used to measure the infectivity. The locations of the peptides may be found in Figure 2.*

**TABLE 5:** Summary of statistical data for the reaction of Ac-NHS with purified PrP^Sc^.

**A**								
**Ac-NHS**	**Inf**	**PGG**	**NKP**	**MKH**	**QHT**	**GEN**	**VVE**	**ESQ**
Slope	−0.04	−0.08	−0.09	−0.09	−0.1	−0.06	−0.04	−0.05
Intercept	−0.27	0.01	−0.15	−0.36	−0.08	−0.01	−0.01	0.08
*R*^2^	0.77	0.999	0.964	0.754	0.99	0.997	0.989	0.953
*p*-value	*p* < 0.05	*p* < 0.01	*p* < 0.01	*p* < 0.06	*p* < 0.01	*p* < 0.01	*p* < 0.01	*p* < 0.01
**B**								
**Ac-NHS**	**Inf**	**PGG**	**NKP**	**MKH**	**QHT**	**GEN**	**VVE**	**ESQ**

Inf	–	*p* < 0.03	*p* < 0.03	*p* > 0.18	*p* < 0.01	*p* > 0.14	*p* > 0.81	*p* > 0.76
PGG		–	*p* > 0.4	*p* > 0.74	*p* < 0.04	*p* < 0.01	*p* < 0.01	*p* < 0.01
NKP			–	*p* > 0.97	*p* > 0.56	*p* < 0.04	*p* < 0.01	*p* < 0.01
MKH				–	*p* > 0.85	*p* > 0.4	*p* > 0.17	*p* > 0.19
QHT					–	*p* < 0.01	*p* < 0.01	*p* < 0.01
GEN						–	*p* < 0.01	*p* < 0.03
VVE							–	*p* > 0.85
ESQ								–
**C**								
**Inf**	**PGG**	**NKP**	**MKH**	**QHT**	**GEN**	**VVE**	**ESQ**	

1 mM	*p* < 0.05	*p* > 0.05	*p* < 0.05	*p* > 0.13	*p* < 0.05	*p* < 0.05	*p* < 0.05	
5 mM	*p* > 0.70	*p* < 0.05	*p* > 0.23	*p* > 0.20	*p* > 0.24	*p* > 0.15	*p* < 0.05	
10 mM	*p* > 0.91	*p* > 0.23	*p* > 0.65	*p* > 0.63	*p* > 0.65	*p* > 0.26	*p* > 0.10	
20 mM	*p* < 0.01	*p* > 0.05	*p* < 0.05	*p* < 0.01	*p* > 0.05	*p* > 0.62	*p* > 0.57	

***(A)** Table of linear regression data (slope, intercept, *R*^2^, *p*-value) relating the change in infectivity (Inf) or the normalized MRM transition signals (log_10_) of the listed peptides for each concentration of Ac-NHS. **(B)***t*-test comparing the slopes of the linear regression from infectivity (Inf) *v*. the normalized MRM transition signal (log_10_) of each listed peptide and the normalized MRM transition signal (log_10_) of each peptide *v*. another peptide. **(C)** Unpaired Student’s *t*-test comparison of the infectivity to the normalized MRM transition signal (log_10_) of a peptide at a specific reagent concentration. p < 0.05 indicate statistical significance. Each PrP^*Sc*^ reaction was performed in triplicate. The infectivity was measured by hamster bioassay (*n* = 6 per reagent concentration). The locations of the peptides may be found in Figure 2.*

The PrP^Sc^ in BH is acetylated to a different extent with Ac_2_O than is the purified PrP^Sc^ by Ac-NHS. At the highest concentration of the reagents (Table 6), the lysines associated with the peptides PGG (*p* < 0.01; K_23_, K_24_, and K_27_), MKH (*p* < 0.01; K_110_), QHT (*p* < 0.03; K_185_), and GEN (*p* < 0.01; K_194_ and K_204_) were acetylated to a significantly lesser extent by Ac_2_O in the BH than by Ac-NHS in the purified preparation. Other peptides, NKP (*p* > 0.95; K_101_, K_104_, or K_106_), VVE (*p* > 0.27; K_220_), and ESQ (*p* > 0.77; K_220_), show no significant differences in the degree of acetylation in either BH or purified PrP^Sc^ at the highest concentration of the reagents. This suggests that K_101_, K_104_, K_106_, and K_220_ share a chemical environment that is not influenced by the molecules present in BH. In contrast, the chemical environments of K_23_, K_24_, K_27_, K_111_, K_185_, K_194_, and K_204_ were more strongly influenced by the molecules present in BH.

**TABLE 6:** Unpaired Student’s *t*-tests comparing the infectivity (Inf) (log_10_), the normalized MRM transition signals (log_10_) of five tryptic peptides (**PGG**WNTGGSR, **QHT**VTTTTK, **GEN**FTETDIK, **VVE**QMCTTQYQK, and **ESQ**AYYDGR), one chymotryptic peptide (**NKP**SKPKTNM), and the normalized Western blot signal from 3F4 epitope (**MKH**M) after the reaction of purified PrP^Sc^ with Ac-NHS and PrP^Sc^-infected brain homogenate with Ac_2_O.

***t*-test**	**Inf**	**PGG**	**NKP**	**MKH**	**QHT**	**GEN**	**WE**	**ESQ**
1	*p* < 0.01	*p* > 0.24	*p* > 0.28	*p* < 0.03	*p* > 0.07	*p* > 0.12	*p* > 0.82	*p* > 0.61
2	*p* > 0.89	*p* < 0.01	*p* > 0.47	*p* < 0.01	*p* > 0.18	*p* < 0.04	*p* > 0.93	*p* > 0.78
3	*p* > 0.21	*p* < 0.01	*p* > 0.09	*p* < 0.01	*p* > 0.47	*p* < 0.01	*p* > 0.80	*p* > 0.12
4	*p* > 0.97	*p* < 0.01	*p* > 0.95	*p* < 0.01	*p* < 0.03	*p* < 0.01	*p* > 0.27	*p* > 0.77

*Row 1: The compared values were from the reactions with 1 mM Ac-NHS and 5 mM Ac_2_O. Row 2: The compared values were from the reactions with 5 mM Ac-NHS and 10 mM Ac_2_O. Row 3: The compared values were from the reactions with 10 mM Ac-NHS and 20 mM Ac_2_O. Row 4: The compared values were from the reactions with 20 mM Ac-NHS and 50 mM Ac_2_O. p < 0.05 indicate statistical significance. Each PrP^*Sc*^ reaction was performed in triplicate. Hamster bioassay (*n* = 6 per reagent concentration) was used to measure infectivity. The locations of the peptides may be found in Figure 2.*

### Reaction of Ac_2_O in BH or Ac-NHS With Purified Prions Results in a Similar Loss of Infectivity

The results of the bioassay of the reaction mixtures are summarized in Figures 8, 9 (Inf). The reactions using the three highest concentrations of Ac-NHS reagent (5, 10, 20 mM) show no statistical difference from the reactions using the three highest concentrations (10, 20, and 50 mM) of the Ac_2_O reagent (*p* > 0.89, *p* > 0.21, *p* > 0.97, Table 6). At the highest concentration of either reagent, the loss of infectivity is approximately 10-fold.

### Loss of Infectivity Is Associated With Acetylation of K_220_ in PrP^Sc^

The slopes of the linear regression of the peptides were statistically compared to that of infectivity (*t*-test, Tables 4B, 5B). This analysis showed that of the seven peptides, only the MKH, VVE, and ESQ peptides showed a *p*-Value that precludes the rejection of the null hypothesis when compared to infectivity (Inf) after reaction with Ac-NHS and Ac_2_O. At each concentration of the Ac_2_O reagent, the extent of the reaction (log_10_) of the MKH peptide is significantly different from that of the log_10_ loss of infectivity (Inf, unpaired Student’s *t*-test, *p* < 0.05, Table 4C). The normalized MRM transition signal (log_10_) from the peptides VVE and ESQ show no significant difference (*p* > 0.3) when compared to the loss of infectivity at the highest concentration of the Ac_2_O (Table 4C) or Ac-NHS (Table 5C). Furthermore, there is no significant difference (*p* > 0.3) between the slopes derived from the linear regression of the loss of infectivity and the normalized MRM transition signal (log_10_) for the peptides VVE and ESQ (Tables 4B, 5B). These lines of evidence are true only for the VVE and ESQ peptides. Since the VVE and ESQ peptides are derived from the tryptic cleavage of K_220_, the loss of signal from these peptides is the result of the acetylation of K_220_. This indicates that the extent of acetylation of K_220_ is associated with the observed reduction in infectivity, although an accumulation of effects from the acetylation of the other lysines cannot be excluded.

### Role of N-Terminal Lysines (K_23_, K_24_, and K_27_) in Prion Replication

The reaction of the N-terminal lysines (K_23_, K_24_, and K_27_) is noticeably different in BH than with purified Sc237 PrP^Sc^. In contrast, the N-terminal lysines of rPrP are readily acetylated with either reagent at all concentrations. This is also true when purified PrP^Sc^ is reacted with Ac-NHS. When PrP^Sc^ in BH is reacted with Ac_2_O, the N-terminal lysines are acetylated to a significantly (*p* < 0.05) lesser extent. These results suggest that the N-terminal region is bound to molecules present in BH, but absent in the purified preparation. In any event, once PrP^Sc^ is purified, the interfering molecules are presumably sufficiently removed and the N-terminal lysines are available to react, as can be seen when the purified samples are reacted with Ac-NHS.

## Discussion

The physicochemical properties of Ac_2_O and Ac-NHS permit two independent measures of lysine reactivity. Ac_2_O is more polar (logP of 0.47 vs. −0.57) than Ac-NHS and is more soluble in water (250 mM vs. 30 mM) than Ac-NHS. Since brain homogenate (BH) has higher concentrations of nucleophiles than the purified material, a correspondingly higher concentration of acetylating reagent (50 mM vs. 20 mM) would be required to compensate for their presence. Thus, the more water-soluble reagent, Ac_2_O, was used to react with prions in BH, while the less soluble reagent, Ac-NHS, was used to react with purified prions. Since these two reagents acetylate the lysines in rPrP equally (*vide infra*), they can be used to provide two independent measures of the effect of lysine acetylation on the infectivity of the prion template.

The sequence **MKH**M (MKH) spans the epitope of the 3F4 monoclonal antibody (mAb) which contains K_110_ (Kascsak et al., 1987; Figure 2). Other work demonstrated that acetylation of K_110_ by the N-hydroxysuccinimide ester of acetic acid (Ac-NHS) prevents the binding of 3F4 to this epitope (Silva, 2012). This results in the absence of a signal, since the primary antibody is no longer able to bind to the epitope. This permits the quantitation of K_110_ acetylation using Western blot in place of mass spectrometry. In principle, this approach can be used with other amino acid/mAb combinations, which extends the utility of the many anti-PrP mAbs.

Even though PrP^C^’s lysines are conserved and strongly influence the propagation, once they are part of the PrP^Sc^ template their role in prion propagation is limited. When the PrP^Sc^’s lysines are acetylated the loss of infectivity is approximately 10-fold. This is substantially less than the 1,000-fold loss of infectivity when DEPC is used to ethoxyformylate the histidines of purified Sc237 prions (McKinley et al., 1981). This loss of infectivity from the ethoxyformylation of histidines is reversed by the addition of hydroxylamine (McKinley et al., 1981), which suggests that the loss of infectivity is not due to a perturbation of the PrP^Sc^ template.

In the Spagnolli et al., model, histidines are largely on the outer surface of the β-solenoid (Spagnolli et al., 2019). The proteinase K digestion used to purify those prions results in the removal of the histidines at positions 61, 69, 77, and 85. The five remaining histidines (positions 96, 111, 140, 177, and 187) reside in the PK-resistant core, PrP 27-30 (Oesch et al., 1985). It is therefore likely that DEPC reacts with a histidine on the surface of the Sc237 prion and that amino acids on the outer surface of a β-solenoid can play a significant role in prion replication. Unlike lysines, histidines bind divalent metals such as copper (Hasnain et al., 2001; Jackson et al., 2001). This suggests that the disproportionate loss of infectivity (1000-fold) associated with histidine ethoxyformylation may be the result of the interference with the binding of a cofactor, such as a divalent cation.

K_220_ resides in the C-terminus of PrP^Sc^, which is much more resistant to denaturation (Kocisko et al., 1996; Vazquez-Fernandez et al., 2012). Reaction of recombinant hamster PrP with the reagents showed that K_220_ is not intrinsically less resistant to reaction relative to the other lysines (*vide supra*) in the native PrP conformation. This suggests that, in the prion conformation, K_220_ resides in a chemical environment that prevents the reagents from reacting with it. In other hamster strains K_220_ is more susceptible to reaction, which supports the idea that its prion strain-dependent chemical environment influences the reactivity of K_220_ (Silva et al., 2016). Thus, the lack of reactivity of K_220_ in the Sc237 prion conformation is a feature of that conformation. When K_220_ does react, however, it is likely that the loss of infectivity results from the disruption of the C-terminal structure of Sc237.

In the recent Spagnolli et al. (2019) model of murine PrP 27-30 PrP^Sc^, lysines K_23_, K_24_, and K_27_ are not included in the model, as they are digested by proteinase K to produce PrP27-30. In this murine model, lysines homologous to K_101_, K_104_, K_106_, K_110_, K_185_, K_194_, K_204_, and K_220_ of hamster PrP are on the outer surface of a β-solenoid. Of these lysines, the one equivalent to K_220_ is part of a salt bridge on the outer surface. This suggests a possible explanation for the lower reactivity of K_220_, even if it locates on the outer surface of the β-solenoid. An alternate possibility is that the murine β-solenoid model does not accurately model the Sc237 prion structure and that K_220_ projects into the β-solenoid, which could explain its lack of reactivity. In either case, significant disruption of the Sc237 C-terminal structural region would be expected to impede prion propagation.

In other strains of purified hamster-adapted scrapie (139H, Dy, 22AH, and 22CH), the N-terminal lysines are also less reactive (Silva et al., 2016). DNA and RNA are known to bind to the N-terminal region of PrP (Weiss et al., 1997; Gabus et al., 2001a,b) and the presence of nucleic acids is reduced, but not eliminated, during prion purification (Diringer et al., 1997), so it is possible that higher concentrations of these molecules are responsible for this effect.

Experiments with transgenic mice expressing PrP^C^ devoid of the N-terminal lysines have shown that the presence of these lysines in PrP^C^ is crucial for the conversion of PrP^C^ to PrP^Sc^ (Turnbaugh et al., 2012). By contrast, our results show that the extent of acetylation of K_23_, K_24_, and K_27_ in PrP^Sc^ is statistically different (unpaired Student’s *t*-test; *p* < 0.05) from that of infectivity, which suggests that these lysines, when in the prion conformation, do not significantly influence prion replication. Our results are consistent with known properties of PrP 27−30, which, despite missing approximately 70 amino acids from its N-terminus, remains infectious. Our results and those of the transgenic mice appear to be contradictory. However, N-terminal lysines may have a significant role in facilitating the conformational conversion of PrP^C^ to PrP^Sc^, whereas once PrP^Sc^ is formed they no longer play a significant role. The molecules preventing the reaction of these lysines with Ac_2_O in BH may be associated with this transformation.

We showed that mass spectrometry can be used to quantitate the role of lysines in prion replication. Mass spectrometry-based analysis could be used to quantitate the covalent modification of other amino acids in PrP^Sc^, such as methionine (Silva et al., 2010, 2013) or tyrosine (Gong et al., 2011). Quantitating those covalent modifications would indicate their relative surface exposure, which would provide useful structural information. Thus, this approach can be applied to determining the importance of other amino acids in prion replication and their relative surface exposure in the prion conformation.

This approach could be applied to other protein misfolding diseases, such as Alzheimer’s Disease (AD), Amyotrophic Lateral Sclerosis (ALS), and Parkinson’s Disease (PD), which have prion-like features (Jucker and Walker, 2013; Grad and Cashman, 2014; Giles et al., 2017). For instance, the differences in incubation periods of acetylated amyloid β, tau, or α-synuclein, could be measured by an analogous bioassay (Giles et al., 2017). In this way covalent modification could be an important means of obtaining important conformational information after the misfolded proteins are denatured (Silva, 2014).

## Conclusion

We have demonstrated that lysines play a limited role in prion propagation, once the PrP^Sc^ template is formed. This implies that acetylating lysines does not substantially perturb the PrP^Sc^ structure, which provides insight into the PrP^Sc^ structure and can be used to refine structural models and infer the structural differences that define prion strains.

## Data Availability Statement

The raw data supporting the conclusions of this article will be made available by the authors, without undue reservation.

## Ethics Statement

The animal study was reviewed and approved by the procedures were governed by a protocol that was approved by the Institutional Animal Care and Use Committee of the United States Department of Agriculture, Agricultural Research Service, Albany, CA, United States.

## Author Contributions

CS designed the study and wrote the manuscript. CS, ID, and ME-B performed the experiments and analyzed and interpreted the data. CS and ME-B critically revised the manuscript. All authors contributed to the article and approved the submitted version.

## Conflict of Interest

The authors declare that the research was conducted in the absence of any commercial or financial relationships that could be construed as a potential conflict of interest.

## Footnotes

1 http://www.real-statistics.com/regression/hypothesis-testing-significance-regression-line-slope/

2 http://www.real-statistics.com/regression/hypothesis-testing-significance-regression-line-slope/comparing-slopes-two-independent-samples/

3 https://www.aphis.usda.gov/aphis/resources/permits

4 www.cdc.gov/biosafety/publications/bmbl5

## Abbreviations

PrP: prion protein
PrP^Sc^: infectious PrP isoform
PrP^C^: normal cellular PrP isoform
rPrP: recombinant PrP.
